# Understanding vitiligo through the eyes of a typical patient in the U.S.

**DOI:** 10.3389/fmed.2025.1511344

**Published:** 2025-03-14

**Authors:** Yan Valle, Torello Lotti, Julia Sigova

**Affiliations:** ^1^Vitiligo Research Foundation, New York, NY, United States; ^2^Department of Dermatology, University of Studies Guglielmo Marconi, Rome, Italy; ^3^Department of Neonatology, Faculty of Continued Medical Education of Pirogov Russian National Research Medical University, Moscow, Russia

**Keywords:** vitiligo, patient journey, quality of life, treatment, burden

## 1 Introduction

This paper builds on earlier research on the *Vitiligo Patient Journey* by providing a profile of a typical individual navigating the care landscape—from awareness to diagnosis, treatment, or coping (1). Vitiligo affects roughly 1% of the US population (2), yet the complexities of this journey remain overlooked by medical professionals and the general public.

We have a fairly good understanding of the workload for a dermatologist since the 1970s, with the prognosis of increased workload due to an aging population (3). The average dermatologist in the U.S. functions almost exclusively as a specialist and cares for approximately 120 patients over a 45-h week, depending on the practice setting and location (4).

However, we have just started to understand the experience and quality of life of millions of people suffering from vitiligo, a lifelong, typically progressive condition with periods of disease erratic activity and remission. This paper aimed to offer insights into the life of a typical vitiligo patient for dermatology professionals, policymakers, and the general public.

## 2 A “typical” vitiligo patient

### 2.1 Individual profile

Meet “Emily,” a representative example of a Caucasian female in her late 20s in the U.S., named after the most popular girl's name the year she was born. At the age of 26, Emily has battled non-segmental vitiligo for several years, resulting in seven patches covering nearly 15% of her body, most visibly located on her face and neck (5).

The etiology of Emily's vitiligo, a chronic autoimmune condition that causes random white patches on the skin, remains elusive. It is influenced by genetic and environmental factors, emotional distress, and possibly mild skin trauma that may have triggered the Koebner phenomenon. Emily's general health is a testament to her strong spirit, with a touch of anxiety. Despite a known family history of thyroiditis, Emily is not aware of any vitiligo cases in her immediate family, and she suffers only from minor health complications such as sleep disturbance and dry eyes (6).

### 2.2 Phenotypic variations and cultural factors

The experience of vitiligo varies significantly by ethnicity, disease severity, and lesion location (7). While Emily's U.S. context provides some legal protection in the workplace, in other cultures, vitiligo is often regarded as a barrier to forming interpersonal relationships, including marriage and employment, compounding the psychological burden (8).

Patients in Africa, Asia, and the Middle East frequently face additional challenges shaped by societal and religious beliefs (9). For instance, in sub-Saharan Africa, vitiligo is sometimes confused with leprosy, resulting in severe social stigmatization. In India, vitiligo can significantly impact marriage prospects, especially for women. In Iran, some women with vitiligo reported that their condition was viewed as “punishment by God for sins or spiritual impurity.”

### 2.3 Diagnosis and treatment journey

In her teens, Emily endured a 3-month delay before consulting a physician, who informed her that her condition was “nothing to worry about,” leaving her in limbo for years. On average, vitiligo patients obtain a formal diagnosis after a mean (SD) of 2.4 (4.1) years, which reflects the U.S.'s 37% misdiagnosis rate, which is lower than Europe's 45% and Africa's 56% (10).

Initially turning to online resources for information, Emily experimented with five self-prescribed treatments, experiencing minor, transient successes accompanied by occasional adverse effects. Frustrated with the daily 40-min makeup regimen to hide her lesions, she finally sought professional help. After consulting three doctors over 18 months, Emily found a dermatologist whose empathy matched their expertise—a significant breakthrough in a realm where 46% of American doctors, and even higher percentages in Europe and Africa (65%), consider vitiligo untreatable.

### 2.4 Quality of life

Emily's vitiligo significantly affects her quality of life across emotional, sexual, social, and professional domains. The visible patches on her face and neck have reduced her confidence and heightened self-consciousness, according to the findings of the VALIANT study, where 49% of patients reported similar feelings (11).

In relationships, vitiligo can strain marriages, particularly in cultures with stigmas around the condition. Married women, like Emily, often face more appearance-related concerns than unmarried ones, potentially affecting intimacy. Despite these challenges, many find supportive partners, and the risk of passing vitiligo to children remains generally low unless both parents have the condition (12). Regular skin checkups and screenings for thyroiditis and vitamin D deficiency would become routine for her family.

Vitiligo may also impact her career prospects. While some individuals with vitiligo thrive professionally, many more encounter daily discrimination and challenges, particularly in appearance-sensitive roles. Psychological distress can hinder work performance, but legal protections may apply, as vitiligo might qualify as a disability under employment law.

### 2.5 Treatment and outlook

Currently, Emily is following a treatment plan that includes UV phototherapy, topical medications, and occasional supplements. She is optimistic that her regimen will restore her skin color within a year (13). Even after successful treatment, she may enjoy a remission period for 4 years, a duration that can potentially double with diligent follow-up care. Cognitive behavioral therapy could bolster her ability to cope, given her tendency toward clinical depression.

### 2.6 Financial and social aspects

The financial and social burden of vitiligo treatment can be significant (14). Research indicates that patients might spend up to 5,000 euros for lesion-free skin (in 2009 prices) (15); however, Emily has incurred vitiligo-related expenses of just under $3,500 so far—modest compared to newer drugs costing upward of $20,000 (16). Medicare offers some relief, but private insurers often favor less costly treatments such as corticosteroids before offering financial support.

For Emily, vitiligo is more than a mere skin condition; it is an integral part of her life, influencing her personal and social interactions without defining her. Although it complicates her life and finances and could affect her career, she perceives it more as a social challenge than a disability. In contrast, individuals with darker skin tones often view vitiligo as a significant hurdle, e.g., with Emily's counterparts in Brazil facing severe discriminatory employment practices at the governmental level (17).

## 3 Discussion

Emily's journey offers a glimpse into the broader challenges faced by vitiligo patients. Her experience of delayed diagnosis, inadequate initial care, and the ongoing search for effective treatment reflects the need for a more holistic approach—one that includes both medical treatment and psychological support. While each patient journey is unique, Emily's story highlights the complexities of living with vitiligo and underscores the importance of improving care pathways for those affected.

Exercises such as the previous Vitiligo Patient Journey Map and this current expansion of the topic serve a crucial purpose in educating healthcare professionals and the general public about a disease that affects over two million people in the U.S. alone and close to 100 million worldwide. By providing a detailed, personalized account of a typical patient's experience, we aim to bridge the gap between clinical understanding and the lived reality of vitiligo patients.

In conclusion, while Emily's story provides valuable insights, it represents just one facet of the vitiligo experience. By building upon this work and continuing to map the patient journey, we can contribute to a more inclusive, effective, and compassionate approach to vitiligo care and management worldwide.
